# Behaviour of synthetic musk fragrances in freshwaters: occurrence, relations with environmental parameters, and preliminary risk assessment

**DOI:** 10.1007/s11356-023-30030-9

**Published:** 2023-09-30

**Authors:** Stefano Tasselli, Michela Rogora, Arianna Orrù, Licia Guzzella

**Affiliations:** 1 National Research Council - Water Research Institute (CNR-IRSA), Via del Mulino 19, 20861 Brugherio, (MB) Italy; 2 National Research Council - Water Research Institute (CNR-IRSA), L.Go Tonolli 50, 28922 Verbania, (VB) Italy

**Keywords:** Synthetic musk compounds, Freshwaters, Sediments, Ecological risk assessment, Environmental fate

## Abstract

The aims of this study were to investigate the presence, possible sources, and potential ecological risks of synthetic musk fragrances in freshwaters and sediments of the main tributaries of a deep subalpine lake in Northern Italy. The total musk concentrations ranged from few ng L^−1^ up to values > 500 ng L^−1^, depending on river characteristics: water flow and the presence of wastewater effluents proved to be the main factors affecting fragrance concentrations. The water flow may indeed dilute fragrance input mainly deriving from treated wastewaters. Good correlations (determination coefficients > 0.60) between synthetic fragrances concentrations and parameters related to anthropogenic impacts confirmed this hypothesis: synthetic fragrances were mainly detected in most polluted rivers crossing urbanized areas. Sediment analysis highlighted accumulation of fragrances in this matrix. Concentrations of synthetic fragrances up to 329 ng g^−1^ organic carbon were measured in sediments of the most contaminated rivers Boesio and Bardello, which also show the highest nutrient content. The preliminary environmental risk assessment revealed that present levels of synthetic musk fragrances do not pose any risk to the studied environmental compartments. However, a probable medium risk level was evidenced during the dry season in the most contaminated rivers Boesio and Bardello. For these reasons, small rivers draining urbanized watersheds and affected by wastewater effluents should be considered synthetic musk contamination hotspots that warrant further research.

**Supplementary Information:**

The online version contains supplementary material available at 10.1007/s11356-023-30030-9.

## Introduction

Synthetic musks are a class of organic chemicals produced in large quantities and widely used in various consumer products as cosmetics, personal care, and household products (Homem et al. 2016; Vimalkumar et al. 2021). These chemicals have raised significant concern in recent years due to their potential threat to the environment and the human health (Fromme et al. 2001; Ramos et al. 2015). Between synthetic musks, polycyclic musks (PCMs) as Galaxolide® (1,3,4,6,7,8‑hexahydro‑4,6,6,7,8,8‑hexamethyl‑cyclopenta‑γ‑2‑benzopyran, or HHCB), Tonalide® (6-Acetyl-1,1,2,4,4,7-hexamethyltetralin, or AHTN), and, in minor part, Celestolide® (4-acetyl-6-tert-butyl-1,1-dimethylindan, or ADBI) and Phantolide® (5-acetyl-1,1,2,3,3,6-hexamethylindan, or AHMI) represent the 95% of the total fragrance market worldwide (European Commission 2008a, b). HHCB and AHTN production alone has been estimated about 1 million pounds per year since these compounds are the most widely employed worldwide and, for this reason, they were inserted on the High Production Volume List by the USEPA (Peck et al. 2006). They are compounds with acetylated and highly methylated pyran, tetralin, and indane skeletons, characterized by low production costs and a widespread availability that allowed a possible increase in the future in their global production and consumption (Hua et al. 2022). Even if they are structurally stable and relatively recalcitrant to degradation (Artola-Garicano et al. 2003), biotic and abiotic processes in the environment as enzymatic reactions and photodegradation can generate synthetic musks metabolites and transformation products. Between them, the most commonly found in the environment due to its concentration and stability is HHCB-lactone (HHCB-L), the main metabolite of HHCB (Bester 2004). This compound is generated mainly in wastewater treatment plants (WWTPs) by biological degradation in activated sludge (Tasselli et al. 2021) and during the ozone treatment (Herrera López et al. 2013). Once generated, this compound is more stable and recalcitrant to degradation respect to HHCB and, because of its higher polarity, it can be measured mainly in the aqueous matrix (Bester 2005).

Since PCMs are products directly applied on the human skin, they are not subjected to metabolic alterations during their employment; therefore, with a regular use, large quantity of these compounds may enter unaltered into the environment (Ternes et al. 2004). Synthetic musks are ubiquitous in the environment but they can be mainly detected in aquatic systems (Peck et al. 2006; Upadhyay et al. 2011; Weinberg et al. 2011). In fact, they can enter the aquatic environment directly, for example, through recreational activities in beaches and rivers as swimming and bathing, but also through indirect discharges of WWTP effluents since current wastewater treatments cannot completely remove these contaminants (Villa et al. 2020). Their release and presence with consequent potential bioaccumulation vary between regions and states according to the different usages (Hong et al. 2021). Based on the available literature, PCMs in surface water spread a wide range of concentrations from few ng L^−1^ to several tens of ng L^−1^ (Zhang et al. 2008; Sumner et al. 2010; Wang and Kelly 2017), meanwhile some sampling sites registering concentrations over µg L^−1^ (Fromme et al. 2001; Lee et al. 2010). Higher concentrations of PCMs in surface waters were generally registered in river systems located in anthropized area or subjected to discharges from WWTPs (Zhang et al. 2020).

Due to their physicochemical properties, synthetic musks are lipophilic, persistent, and highly bioaccumulative compounds. They may be easily adsorbed onto organic matter and, in aquatic ecosystems, accumulated in sediments and organisms (Kannan et al. 2005; Subedi et al. 2014; Huang et al. 2016; Vimalkumar et al. 2021). It has been highlighted that sediments are the final sinks for synthetic musks since field-derived log *K*_OC_ (organic carbon–water partition coefficient) values for HHCB and AHTN are in the range of 3.86–4.86 (Fooken 2004; Wang et al. 2018). In fact, once release in the aquatic environment, PCMs can be removed primarily by outflowing wastewaters or losses into the atmosphere, while in summer direct photolysis is the main elimination process especially for AHTN (Buerge et al. 2003). Available literature reports the presence of polycyclic musk in different matrices as wastewater and sludge samples (Sun et al. 2014; Tasselli and Guzzella 2020) but also in surface waters and sediments (Villa et al. 2012; Wang et al. 2018). HHCB and AHTN were observed also in fish and other aquatic organisms especially in water bodies located near urban centers and anthropized areas (Zhang et al. 2013; Lange et al. 2015).

Various toxic effects related to synthetic musks were already highlighted, together with their potential action as endocrine disrupter chemicals (Tumová et al. 2019). Based on published data, polycyclic musks may be considered toxic from ppb to low ppm levels to aquatic invertebrates which, for longer exposure periods, appear to be more sensitive than fish (Brausch and Rand 2011). From these considerations, an assessment of the presence and behavior of synthetic musks in aquatic environments is still crucial, especially in Italy were data regarding PCM concentration in the environment are very scarce, despite Italy was the EU-Member State with the highest detergent consumption according to the last available survey performed by the International Association for Soaps, Detergents and Maintenance Products A.I.S.E. ﻿(HERA 2008). To start filling this gap of knowledge, in this study the presence of polycyclic musk fragrances was assessed for the first time in surface waters and sediments of the main tributaries of a deep subalpine lake in Northern Italy. Besides this, sample compound composition was considered to evaluate possible sources of PCMs, We also assessed the possible relationships between these pollutants and selected physical and chemical parameters of the considered rivers. Ultimately, potential ecological risk of synthetic musks for living aquatic organisms was evaluated according to the measured concentration levels.

## Materials and methods

### Study area

This study was carried out on Lake Maggiore, a deep subalpine lake belonging to the LTER (Long-Term Ecological Research) network (site EU-IT08-001-A). It is the second largest and the second deepest lake in Italy, with a surface area of 213 km^2^, a volume of 38 km^3^ and a maximum depth of 370 m. The catchment area is equal to 6599 km^2^ and, administratively, the area falls within the borders of Italy (3229 km^2^; Piedmont and Lombardy regions) and Switzerland (3370 km^2^; Canton Ticino), even if 80% of the lake surface is in the Italian territory. Studies on Lake Maggiore and its tributaries have been performed continuously since the 1980s in the framework of the limnological campaigns funded by the International Commission for the Protection of Italian-Swiss Waters (CIPAIS; www.cipais.org). Lake Maggiore is a holo-oligomictic lake as it rarely undergoes complete mixing (Guilizzoni et al. 2012), with a theoretical water renewal time of 4.5 years (Ambrosetti et al. 2012). Several other lakes are included in the Lake Maggiore watershed as lakes Orta, Lugano, and Varese. The lake watershed is prevalently occupied by mountains with low population density, apart from the shoreline area where towns like Varese, Lugano, Bellinzona, and Verbania are located. The total number of the inhabitants living in the Lake Maggiore area is 640,000, approximately. Especially in summer, tourism accounts for more than 300,000 additional equivalent inhabitants. Lake Maggiore hydrographic basin has Ticino Tributary, Toce, and Tresa as the main tributaries, while the only emissary is the Ticino River. Among other minor tributaries, considered in this work, there are Boesio River, which flows into the lake at Laveno-Mombello, Bardello River, emissary of Lake Varese, and Margorabbia River, which flows into Tresa River before entering the lake near the city of Luino. The coastal water environment is directly influenced by the contributions of tributaries and discharges into the lake; in fact, the quality of coastal waters can be very different from that of the pelagic zone. Different types of industries are located in this area which, together with urban WWTPs, can create serious problems in the absence of a correct and effective water management (Marziali et al. 2021).

### Sampling design

**Fig. 1:**
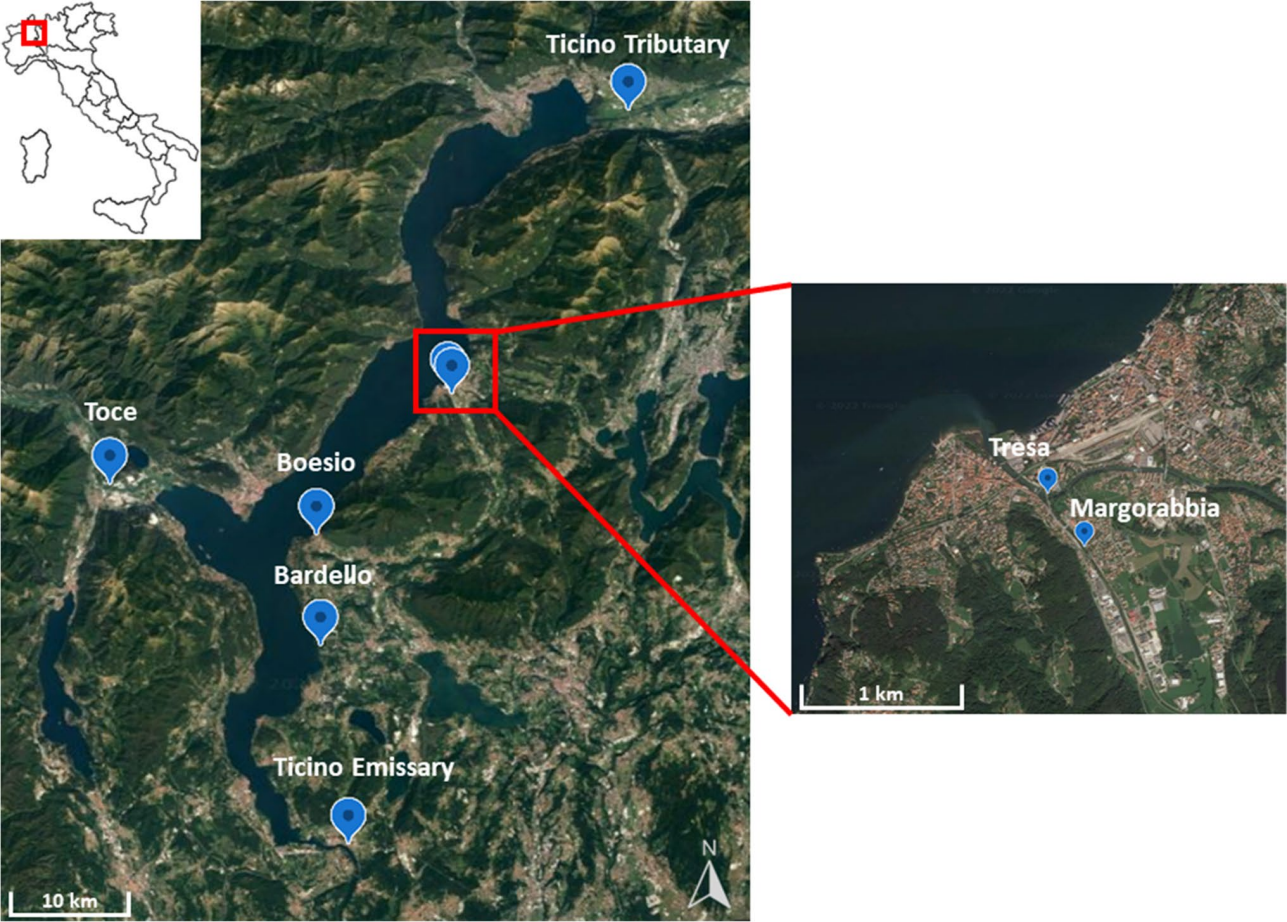
Sampling locations of Lake Maggiore tributaries

In this study, water and sediment were sampled at the rivers’ mouth of Ticino Tributary, Toce, Tresa, Margorabbia, Boesio, Bardello, and Ticino Emissary (Fig. 1). Instantaneous water samples were taken monthly almost at the same time of the day, from April, 2021 to March, 2022, while sediments were sampled three times during the year (*n* = 3), in July and October 2021 and April 2022. The pluviometric regime in Lake Maggiore area is usually characterized by two maxima in spring and autumn and two minima in winter and summer (Saidi et al. 2013). However, within the study period (2021–2022), precipitation showed a quite distinct pattern, with higher than average precipitation (and consequently river discharge) in July, 2021, and very scarce precipitation during the first part of 2022. As a consequence sediment sampling covered sharply different hydrological conditions, from very wet in July, 2021 (mean precipitation 335 mm with respect to the long-term average 1978–2020 for this month of 132 mm) to moderately dry in April, 2022 (99 mm, with respect to 164 mm) (CNR IRSA 2022, 2023).

Synthetic musk fragrances were measured in instantaneous freshwater samples of Lake Maggiore main tributaries every month for 1 year (*n* = 12). During the first 6 months, from April to September, 2021, fragrances were measured in all the seven main tributaries while, from October, 2021, to April, 2022, the analysis of fragrances was not carried out in Ticino Emissary and Toce rivers. Water samples were taken using a bucket and water was then stored in 2.5-L amber glass bottle, previously washed with acetone, and preserved at 4° C until analyses for polycyclic musk, carried out the day after samplings. One liter of water was also collected in polyethylene bottles for chemical analyses which were completed within 3 days after the sampling. All materials were previously rinsed with the respective river water before use.

For sediments, depositional areas were identified at each site, and subsamples of freshly deposited sediments (5–10 cm depth) were collected using a stainless-steel spoon and mixed to obtain a 2-L representative sample. Sediments were preserved in dark glass bottles at 4 °C until freeze-drying within 1 week from sampling. Dry sediments were then sieved to separate the finest fraction (< 63 µm grain size), in which organic pollutants usually accumulate, for chemical analyses (US EPA 2001; OSPAR 2002).

### Chemicals

Polycyclic musk standards HHCB (purity 95%), HHCB-L (purity 97%), AHTN (purity 98%), ADBI (purity 98%), and AHMI (purity 98%) were purchased from Spectra 2000 (Rome, IT). Deuterated internal standard AHTN-D_3_ was purchased from LGC Standards (Manchester, USA). A mix working solution was prepared using standards to obtain a final concentrations of 40 ng μL^−1^ for HHCB and HHCB-L and 8 ng μL^−1^ for ADBI, AHMI, and AHTN according to relationship between compounds previously evaluated for Italian wastewaters (Tasselli and Guzzella 2020; Tasselli et al. 2021). AHTN-D_3_ was diluted in acetone for water analyses and in *n*-hexane for sediment samples to a final concentration of 1 ng μL^−1^. Solutions were stored at − 30 °C in 25 mL flasks in the dark to prevent photolysis. Employed solvents were all obtained from VWR International with analytical grade (Radnor, PA, USA).

### Water and sediment analysis

Water samples were analyzed using method already published in (Tasselli and Guzzella 2020), slightly modified for freshwater samples. Briefly, 0.5 L was filtered using glass fiber filters (nominal pore of 0.7 μm) before adding 25 ng of deuterated internal standard AHTN-D_3_. Samples were then reversed solid-phase extracted using C_18_ cartridges (Avantor™ BAKERBOND™, Radnor, PA, USA) and eluted with *n*-hexane:dichloromethane 1:3 *v*/*v* and *n*-hexane:dichloromethane 1:1 *v*/*v*. Samples were then concentrated at 30 °C using N_2_ flux (MultiVap 10, LabTech, Italy) to 0.5 mL and transferred in micro-vials for GC–MS/MS analysis.

Samples were also analyzed for pH, conductivity (potentiometric methods), alkalinity (Gran’s titration), reactive and total phosphorus, N–NH_4_, total nitrogen, reactive silica (UV–VIS spectrophotometry), N–NO_3_ (ion chromatography), total organic carbon (TOC) (high-temperature catalytic combustion) using standard methods for freshwater samples (APHA, AWWA, WEF, 2012; APAT, IRSA-CNR 2003). These analyses were not performed for the Margorabbia River.

Sediment samples of 0.4 g were extracted with *n*-hexane:acetone 3:1 *v*/*v* using a hot-Soxhlet apparatus (Büchi B-811 Flawil, Switzerland), concentrated using N_2_ flux at 0.1 mL, solvent-exchanged in 1 mL acetone, and resuspended in 200 mL of ultrapure water. Samples were then processed according to method descripted for water samples.

Details about analytical method in GC–MS/MS can be found in Tasselli and Guzzella (2020).

For each sediment sample, organic carbon content was determined in 0.2–0.5 g d.w. samples by back-titration after oxidation with potassium dichromate in the presence of sulfuric acid according to Walkley and Black (Schumacher 2002).

### Quality assurance and quality control

Methods for PCM analyses in water and sediment samples were validated based on the procedure descried by EuraChem (Magnusson and Örnemark 2014). Linearity, limits of detection, precision, and accuracy were evaluated. Linearity was checked by injection of six standard samples containing all PCMs at different concentration levels. Limit of detection was calculated based on the signal-to-noise ratio (*S*/*N*) of individual peaks, assuming a ratio of 3:1. Since certified reference materials are not available for these chemicals, precision and accuracy were evaluated by spiking real water and sediment samples in triplicate at different concentration levels to cover the entire concentration range of the analyzed samples. Details about validation parameters can be found in Supplementary Materials (Tables S1 and S2). Due to the widespread use of PCMs, precautions were employed to prevent laboratory contamination. Laboratory personnel avoided the use of scented personal care products, and all glassware was acetone washed prior to using it. Procedural blanks were analyzed for every extraction batch of water and sediment and all samples were blank subtracted since trace levels of HHCB, HHCB-L, and AHTN were detected.

### Preliminary environmental risk assessment of HHCB and AHTN

The environmental risk assessment of HHCB and AHTN in surface water and sediment was performed according to the hazard quotient (*HQ*) approach (Raybould et al. 2011). *HQ* represents the ratio of the potential exposure to a substance and the level at which no adverse effects are expected. In this work, the potential environmental risk posed by PCMs in freshwaters and sediments was assessed by calculating the *HQ* according to Eq. (1):

$$HQ=\frac{MEC}{PNEC}$$

where *MEC* refers to the measured environmental concentration; and *PNEC* refers to the predicted no effect concentration. According to European Union Risk Assessments, *PNEC* values for HHCB and AHTN were 4400 ng L^−1^ and 2800 ng L^−1^ for water and 1970 ng g^−1^ d.w. and 1720 ng g^−1^ d.w. for sediment (European Commission 2008a, b). PNEC values were calculated from results of prolonged toxicity tests carried out on algae, the invertebrates *Daphnia magna* and *Acartia tonsa*, and fish for water and regarding midge larvae, amphipods, and worms regarding sediments. For both matrices, an assessment factor of 10 was applied for PNEC calculation. Overall, *HQ* values > 1.0 indicate that a high risk is expected, while values between 0.1 and 1.0 indicate a medium risk, and values < 0.1 indicate a low risk (Sánchez-Bayo et al. 2002). The combined ecological risk was also calculated by the sum of the individual *HQ*_*i*_ of each PCM (Guo et al. 2013).

### Statistical analysis

Statistical analyses were carried out using R software (version 4.2.0) and Past (4.03). The Shapiro–Wilk test was used to verify the data normality while the homogeneity of variances was evaluated using the Levene test. In case of significance (*p* < 0.05), non-parametric statistical tests were employed. In particular, the Kruskal–Wallis rank sum test and Dunn’s post-hoc test were performed to compare fragrance concentrations in the various tributaries, while Spearman correlation analysis was employed to evaluate possible relationships between concentrations of different fragrances and river chemical parameters. Map of sampling sites was created using Google Earth version 7.3.6.9345.

## Results and discussion

### Concentrations and spatial distribution of PCMs in river systems

#### Water samples

Results concerning fragrance quantification in freshwater samples are here summarized. Ticino Tributary showed always total PCM concentrations < LOD while in River Toce only HHCB was detected at concentrations below 20 ng L^−1^. We hypothesized that the measured very low concentrations in these rivers were due to a combination of a lower contamination level of their waters with respect to the other tributaries and to their high water flow (Fig. S1) which can dilute PCMs to values below the limit of detection. Since these tributaries did not significantly contribute to the apport of synthetic fragrances at Lake Maggiore basin even during the summer period, characterized by seasonal tourism in those areas, their analysis was not continued in the following period.

**Fig. 2:**
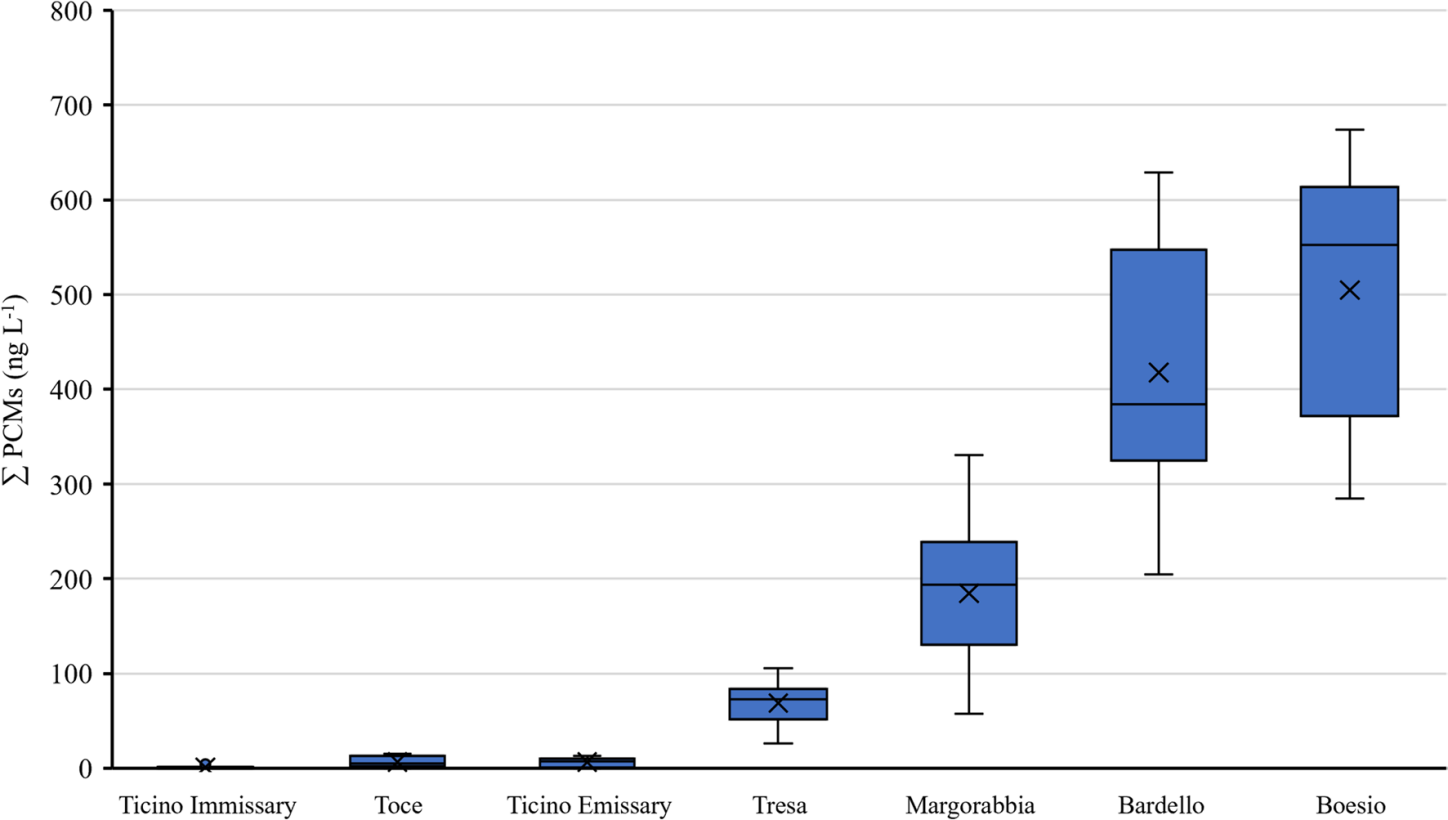
Boxplots of total polycyclic musk concentrations measured in freshwater samples of Lake Maggiore tributaries. Each boxplot shows minimum, maximum, 5th and 95th percentiles, mean, and median values

Considering other tributaries, detected PCM concentrations vary significantly between rivers (Fig. 2). Boesio and Bardello rivers were the most polluted by PCMs, with mean concentrations of 504 ± 130 ng L^−1^ and 417 ± 137 ng L^−1^, respectively. These rivers, characterized by a modest discharge (Fig. S1), cross heavily anthropized areas. From the inlet to the mouth, these rivers receive numerous discharges of wastewaters, both of civil and industrial types which contribute to accentuate the poor quality of the waters (ARPA 2013; Mosello et al. 2001) and could represent punctual sources of synthetic fragrances release into the environment, as demonstrated in previous studies (Tasselli and Guzzella 2020; Tasselli et al. 2021). For example, the WWTP of the city of Casalzuigno with its effluent located along Boesio River or the WWTP of the city of Gavirate with its effluent located along the Bardello River (ARPA 2013).

Margorabbia River showed lower and less variable levels of synthetic musk, registering mean concentrations of 184 ± 74 ng L^−1^. A mean concentration of 68 ± 23 ng L^−1^ and 6 ± 4 ng L^−1^ of total fragrances was instead measured at Tresa and Ticino Emissary rivers. The low concentrations of fragrances detected in these rivers might be due to a dilution effect caused by the lakes from which they originate, Lake Ceresio and Lake Maggiore, respectively. Statistically significant differences were evidenced between Ticino Emissary and Bardello (*p* < 0.001), Boesio (*p* < 0.001), and Margorabbia (*p* < 0.05) rivers; on the other hand, no differences were found between Bardello, Boesio, and Margorabbia rivers, and between Tresa and Ticino Emissary rivers (*p* > 0.05). Regarding Margorabbia River, the presence of a municipal WWTP effluent along the river course, which may introduce synthetic fragrances, is documented as well (ARPA 2013) and could therefore explain the slightly higher concentrations measured in this river before its confluence with River Tresa, a few hundred meters before flowing into Lake Maggiore.

**Fig. 3:**
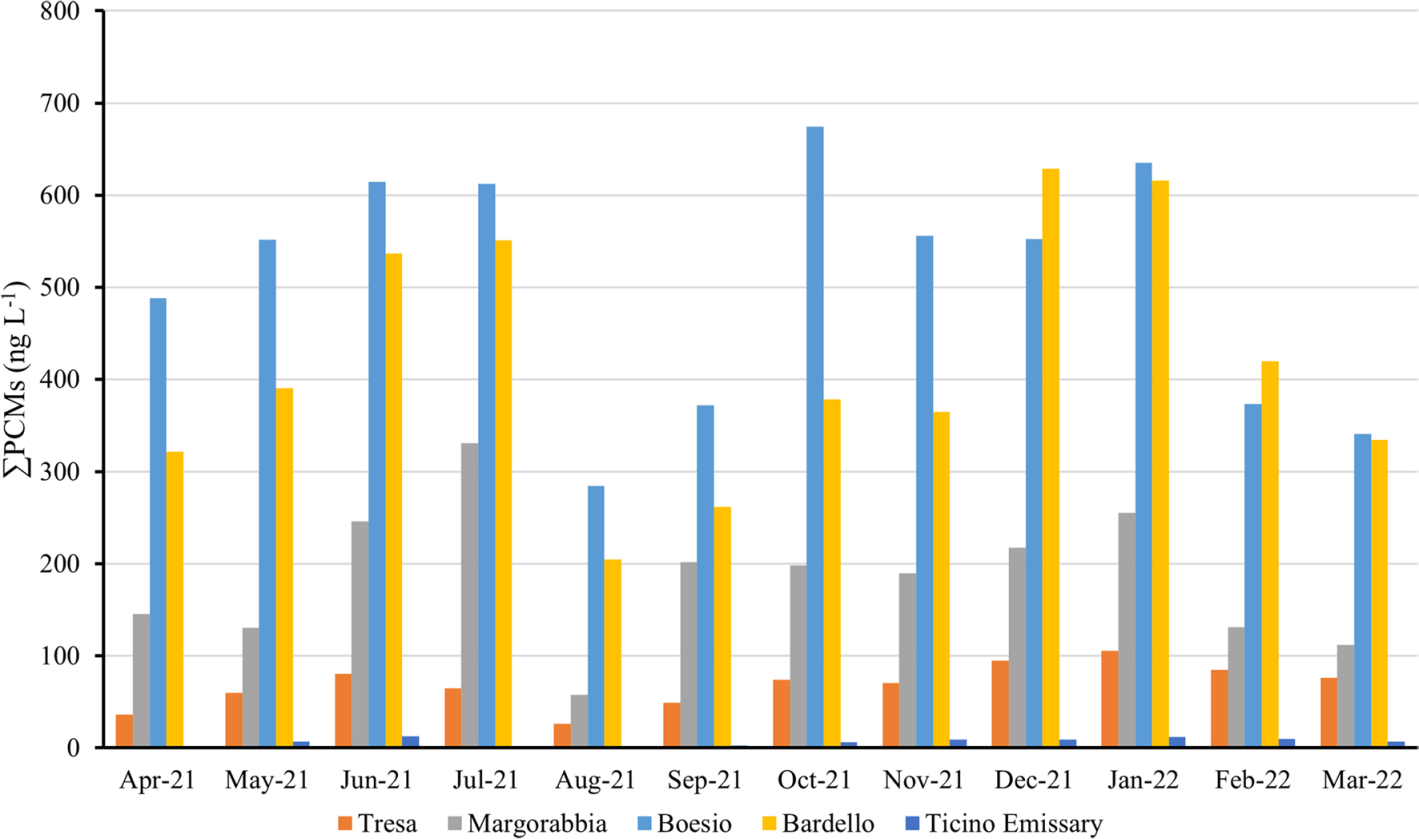
Temporal trends of total polycyclic musk concentrations measured in freshwater samples of Lake Maggiore tributaries

Figure 3 shows monthly values of the total fragrances in the water samples of the study rivers.

In general, a similar pattern was evidenced for all the rivers, with higher concentrations in June–July and December–January, even if the minimum concentration of fragrances was measured in all rivers during August, 2021. This result agrees with the hypothesis of dilution already demonstrated for wastewaters (Tasselli et al. 2021): in fact, sampling during August, 2021, was performed after 1 month of high river discharge (Fig. S1) as an effect of quite heavy rainfall in July, 2021, which may have diluted PCM concentrations. Maximum concentration was instead measured in July, 2021, at Margorabbia (331 ng L^−1^) before the rain events, in October and January, 2022, at Boesio (674 ng L^−1^ and 635 ng L^−1^), in December, 2021, at Bardello (629 ng L^−1^) and in January, 2022, at Tresa (106 ng L^−1^). Also by considering the entire annual pattern, Boesio and Bardello were confirmed as the most contaminated rivers by synthetic fragrances.

#### Sediments

**Fig. 4:**
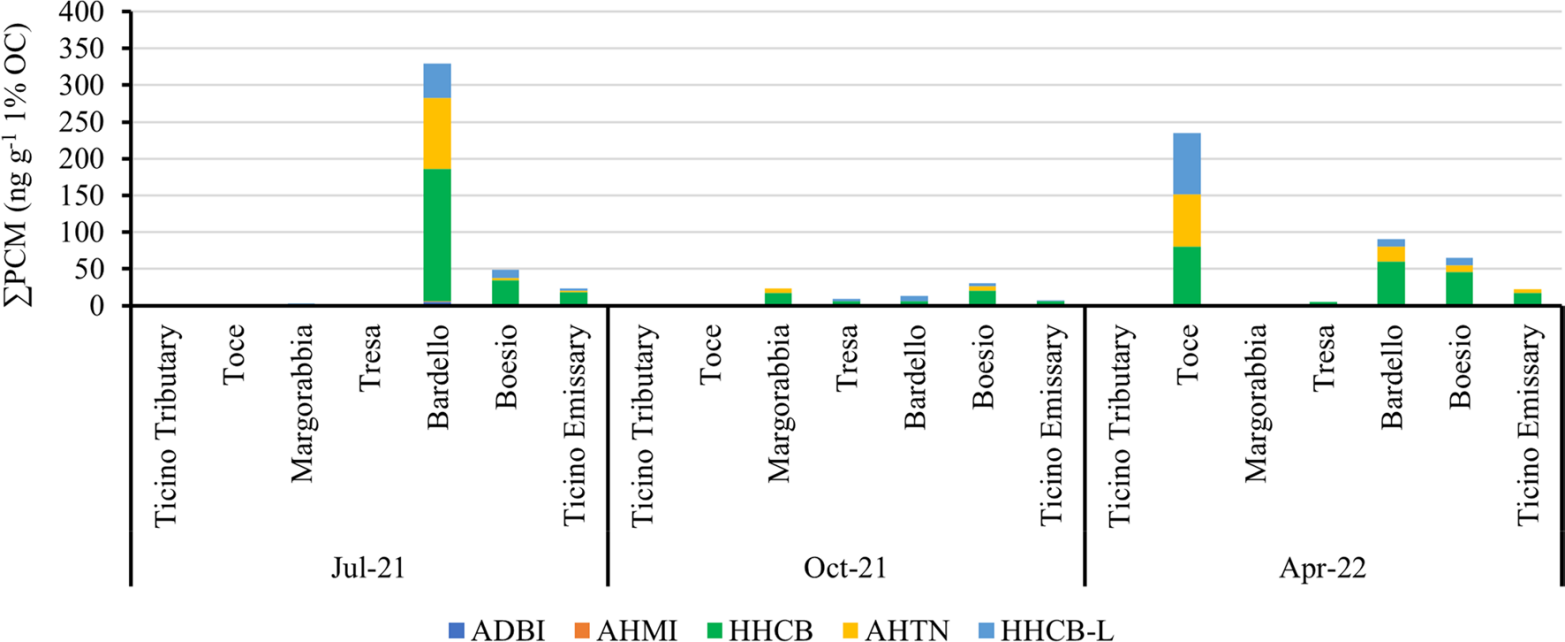
PCM concentration (ng g^−1^ 1% O.C.) in Lake Maggiore tributaries sediment samples

Due to their hydrophobic nature, organic compounds are mainly adsorbed on sediment organic matter so that the normalization of data on organic carbon (OC) content leads to comparison between sites with different OC (Pisanello et al. 2016). For this reason, total OC was measured for each sediment samples and concentration of PCMs was normalized to this parameter. Figure 4 shows PCM concentration OC normalized in each sediment samples of Lake Maggiore tributaries analyzed during the three sampling campaigns.

In Ticino Tributary the analyzed compounds were always < LOD, while in Toce River 235 ng g^−1^ OC of total synthetic fragrances were detected only in April 2022. Concentrations < 25 ng g^−1^ OC were observed in Margorabbia and Tresa rivers both in July and October, 2021, while in April, 2022, total synthetic fragrances in Margorabbia River were below the detection limit. The minimum total PCM concentrations were found for almost all rivers in October, 2021. Considering the entire sampling year, the maximum concentration of total PCMs was measured in the sediments of Bardello River (329 ng g^−1^ OC) in July, 2021, which could be considered the most contaminated river by polycyclic musk fragrances. In this case, also the contribution probably deriving from the WWTP effluent of the city of Gavirate may have determined the high concentrations measured in the river. As for water samples, synthetic fragrances in sediments seemed to be influence by the respective river water flow. In fact, higher concentrations were measured in all tributaries during July, 2021, and April, 2022, after periods of scarce rain events and modest water flow especially for Toce and Bardello rivers (Fig. S1), while in October, 2021, the concentration of synthetic fragrances decreased in all rivers probably due to a dilution effect caused by the higher mean flow rate registered in all rivers during months prior to sampling.

### Composition and origin of PCMs in rivers

**Fig. 5:**
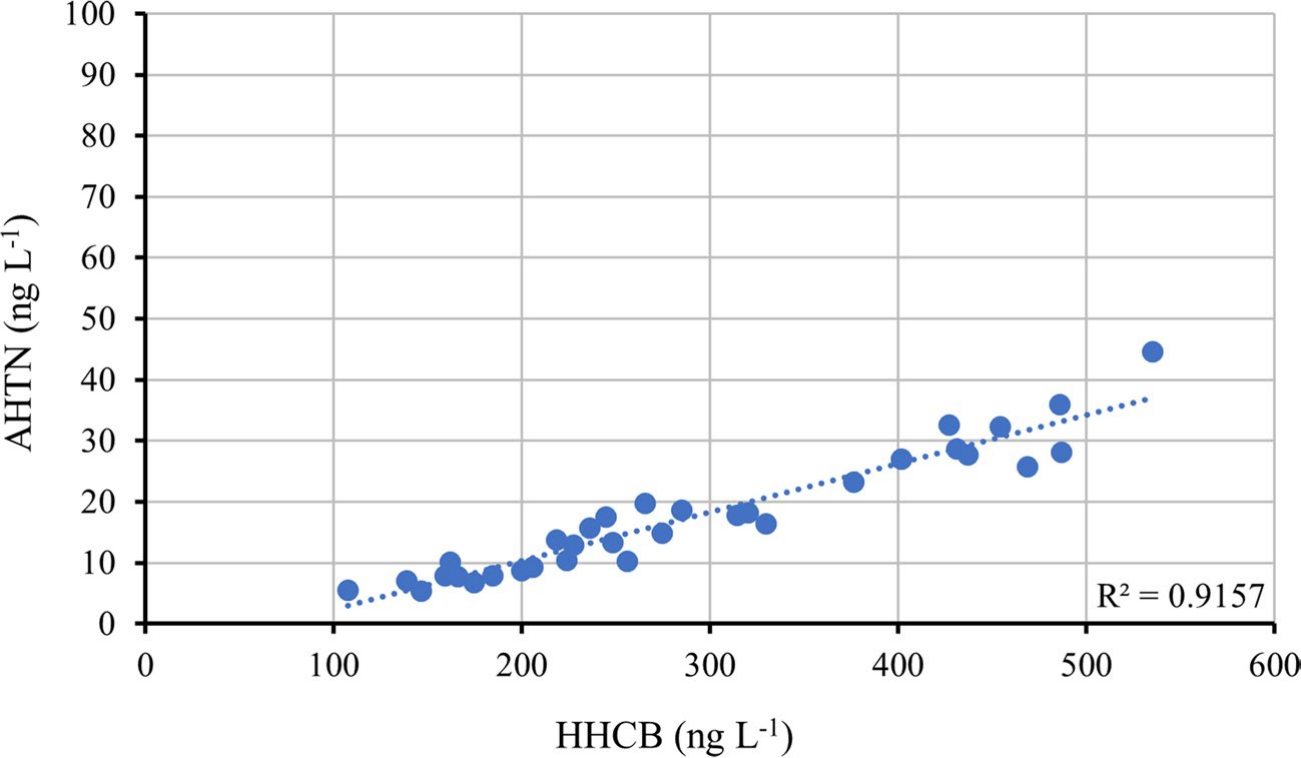
HHCB/AHTN ratios in surface water samples with HHCB and AHTN values > LOD

In all the tributaries of Lake Maggiore ADBI and AHMI were always lower than the LOD value, except in few samples of Boesio and Bardello rivers, where ADBI was detected at trace levels with concentrations always below 1 ng L^−1^. HHCB was the main measured compound in all freshwater samples and always represented above the 70% of the total PCMs in the samples. In rivers located on the more anthropized shore, Tresa, Margorabbia, Boesio, and Bardello, a low contribution of HHCB-L was measured, with values in the range 12–26% while AHTN contributed only for < 5% of the total PCMs in the abovementioned rivers except Tresa. In Ticino Emissary, almost all fragrances detected in water samples were represented by HHCB. This type of profile regarding synthetic fragrances generally agrees with their use in commercial products in which HHCB and AHTN are the main utilized compounds (Clara et al. 2011). The low concentrations of AHTN respect to HHCB may also be explained by a photochemical degradation process: in fact, Sanchez-Prado et al. (2004) evaluated under laboratory conditions the degradation of six polycyclic musk under UV irradiation measuring high degradation rates for AHTN in respect to HHCB and even (Buerge et al. 2003) carried out various UV-irradiation experiments using lake water samples and measured half-lives of 4 h for AHTN and 135 h for HHCB. Regarding sediments, HHCB was the main detected polycyclic musk (Fig. 4), as expected from its chemical properties as hydrophobicity (K_OW_ = 5.3), while the contribution of its metabolite HHCB-L was lower than in water samples, since this compound is characterized by a higher polarity. The relation between HHCB and AHTN was further analyzed in all surface water samples of this study and a coefficient of determination (*R*^2^) of 0.91 was obtained (Fig. 5). Spearman correlation test expressed high significance between the two chemicals (*ρ* = 0.95; *p* < 0.001), supporting the hypothesis of the same origin of these compounds. HHCB/AHTN ratios were then calculated for each sample and results were compared to those already measured by Tasselli et al. (2021) in wastewater samples: the Mann–Whitney U test resulted not significant (*p* = 0.35), thus revealing that synthetic fragrances measured in surface waters may derive from outflows of wastewater treatment plants as previously evidenced in other studies worldwide (Horii et al. 2007; Zhang et al. 2020; Heredia et al. 2023).

### Relations between fragrance occurrence and chemical parameters

The hypothesis of WWTP effluents as possible sources of synthetic fragrances measured in surface waters was further assessed by considering other chemical variables measured in the same rivers and periods. Mean values of the main chemical parameters measured in Lake Maggiore main tributaries and emissary are shown in Table 1 while detailed monthly data are reported in Supplementary Materials (Fig. S2).

**Table 1:** Mean values and standard deviations of the main chemical parameters measured in Lake Maggiore main tributaries and emissary during the study period

*River*	*Cond*^*a*^* (µS cm*^*1*^*)*	*Alc.*^*b*^* (meq L*^*−1*^*)*	*N-NO*_*3*_^*c*^* (µg L*^*−1*^*)*	*N-NH*_*4*_^*d*^* (µg L*^*−1*^*)*	*N org*^*e*^* (µg L*^*−1*^*)*	*TN*^*f*^* (mg L*^*−1*^*)*	*TP*^*g*^* (µg L*^*−1*^*)*	*RSi*^*h*^* (mg L*^*−1*^*)*	*TOC*^*i*^* (mg L*^*−1*^*)*	*n*
*Ticino Tributary*	211.2 ± 66.3	0.9 ± 0.3	652.3 ± 174.4	11.0 ± 5.8	88.3 ± 25.7	0.8 ± 0.2	6.8 ± 1.9	2.3 ± 0.4	0.6 ± 0.13	6
*Toce*	166.6 ± 51.1	0.8 ± 0.1	458.7 ± 75.1	27.8 ± 10.1	91.8 ± 26.6	0.6 ± 0.1	16.8 ± 4.0	2.1 ± 0.2	0.5 ± 0.1	6
*Tresa*	227.0 ± 21.5	2 ± 0.2	1170.4 ± 148.1	101.8 ± 65.9	204.8 ± 67.1	1.5 ± 0.2	29.7 ± 7.4	0.8 ± 0.3	1.5 ± 0.2	10
*Boesio*	630.2 ± 122.4	5.0 ± 0.8	4465.1 ± 565.5	142.8 ± 115.3	628.4 ± 380.3	5.3 ± 0.7	304.5 ± 94.6	3.2 ± 0.6	2.9 ± 0.5	10
*Bardello*	372.4 ± 78.9	3.1 ± 0.7	1683.6 ± 327.6	178.8 ± 161.6	527.1 ± 176.6	2.4 ± 0.5	168.2 ± 56.0	1.9 ± 0.5	3.2 ± 0.4	10
*Ticino Emissary*	145.1 ± 5.5	0.9 ± 0.1	579.0 ± 99.3	16.5 ± 8.6	185.2 ± 86.0	0.8 ± 0.1	10.6 ± 2.2	0.7 ± 0.5	1.1 ± 0.2	10

a, conductivity at 20 °C; b, alkalinity; c, nitrate; d, ammonium; e, organic nitrogen obtained by difference between total nitrogen and inorganic nitrogen (N–NH_4_ and N–NO_3_); f, total nitrogen; g, total phosphorus; h, reactive silica; i, total organic carbon. December 2021 and January 2022 samplings were excluded from elaborations as they were carried out, for technical reasons, on different dates than the measurement of fragrances. Chemical analyses were not carried out on River Margorabbia

According to these data, Bardello and Boesio are characterized by markedly higher concentrations of solutes (higher conductivity and alkalinity) and P and N compounds than the other rivers. In particular, TP and organic nitrogen concentrations indicate a higher level of contamination of Bardello and Boesio by untreated effluents. Previous studies and monitoring programs have highlighted the scarce water quality of these two rivers, due to various sources of pollution along their water course, from untreated domestic and industrial sewage to WWTP discharge (Guzzella et al. 2008; Corno et al. 2023; CNR IRSA 2019). In addition, River Bardello is the outflow of the highly eutrophic Lake Varese (Dresdi et al. in press). From these data it can be deduced that fragrances are more present in rivers affected by higher nutrient organic matter content. Possible relationships between concentrations of polycyclic musks measured in water and the main chemical parameters were further investigated and details are summarized in Table 2.

**Table 2:** Spearman’s correlation analysis between polycyclic musk concentrations and chemical parameters of freshwater samples

*Var1*	*Var2*	*ρ*	*p-value*
*PCM (ng L*^*−1*^*)*	Cond (µS cm^−1^)	0.89	2.398E − 14
*PCM (ng L*^*−1*^*)*	Alc. (meq L^−1^)	0.88	8.749E − 14
*PCM (ng L*^*−1*^*)*	NO3 (µg L^−1^)	0.91	8.882E − 16
*PCM (ng L*^*−1*^*)*	NH4 (µg L^−1^)	0.69	9.944E − 07
*PCM (ng L*^*−1*^*)*	TN (mg L^−1^)	0.90	1.776E − 15
*PCM (ng L*^*−1*^*)*	N org (µg L^−1^)	0.69	4.698E − 06
*PCM (ng L*^*−1*^*)*	TP (µg L^−1^)	0.89	1.132E − 14
*PCM (ng L*^*−1*^*)*	Rsi (mg L^−1^)	0.84	1.010E − 11
*PCM (ng L*^*−1*^*)*	TOC (mg L^−1^)	0.75	3.230E − 08

Polycyclic musk fragrances are positively correlated with chemical parameters related to solute concentrations in river water (alkalinity and conductivity), which are markedly higher in Boesio and Bardello rivers than in the other tributaries (Table 1). PCMs are also positively related with TP and N compounds which are indicators of nutrient enrichment due to contamination by untreated wastewater. Many studies relate the occurrence of polycyclic musk fragrances with anthropogenic impacts as human density and activities. For example, in China and Korea synthetic fragrances were demonstrated to be released in the environment through WWTP effluents since high concentrations were detected in proximity of these areas (Lee et al. 2010; Hu et al. 2011). Synthetic musk fragrances were detected even in Indian (Vimalkumar et al. 2021) and in some European rivers (Bendz et al. 2005; Sumner et al. 2010; Villa et al. 2012; Lange et al. 2015; Homem et al. 2016) in which human activities and the presence of discharges were identified as the main emitting sources. Generally, as demonstrated even in our study, dilution is the main mechanism affecting PCM concentration in surface waters (Lee et al. 2010; Chase et al. 2012; Vimalkumar et al. 2021). The farther is the WWTP effluent point from the sampling station and/or the greater is the water flow rate of the river itself, the lower is the measured fragrance concentration.

**Fig. 6:**
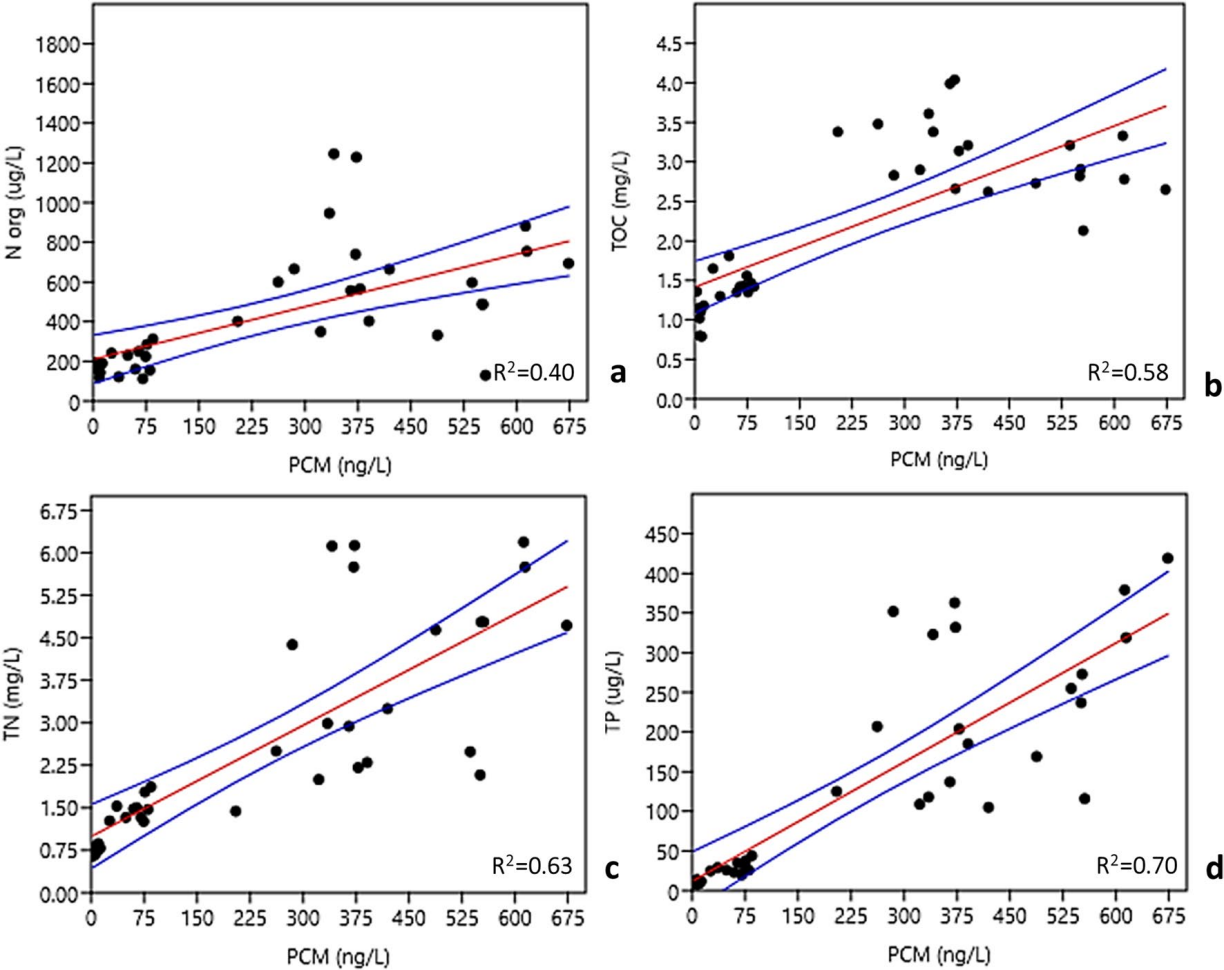
Linear regressions between polycyclic musk fragrances (PCM) concentrations (ng L^−1^) and the respective values of **a** N org (µg L^−1^); **b** TOC (mg L^−1^); **c** TN (mg L^−1^); **d** TP (ug L^−1^). Coefficients of determination (*R*^2^) are also reported

Starting from significant correlations derived from Spearman analysis, in Fig. 6 the resulting linear regressions between polycyclic musk fragrance concentrations and main parameters strictly related to anthropogenic impacts are shown.

### Comparisons with other studies

Observed concentrations of HHCB and AHTN in freshwater samples were compared with data available from other studies (Table 3).

**Table 3:** Worldwide concentrations of HHCB and AHTN in surface waters (ng L^**−**1^)

Location	HHCB (ng L^−1^)	AHTN (ng L^−1^)	Reference
Asia			
Nakdong River, Korea	100–13,920	30–2800	Lee et al. (2010)
Suzhou Creek, China	20–93	8–20	Zhang et al. (2008)
Haie River, China	3.5–32	2.3–26.7	Hu et al. (2011)
Songhua River, China	< LOD-37	< LOD-8	Lu et al. (2015)
America			
Hudson River, USA	3.95–25.8	5–22.8	Reiner and Kannan (2011)
Europe			
Rivers near Berlin, Germany	70–1590	20–530	Fromme et al. (2001)
River Ruhr, Germany	< LOD-600	< LOD-120	Bester (2005)
River Ammer, Germany	1–260	1–60	Lange et al. (2015)
Höje River, Sweden	30–1580	n.d	Bendz et al. (2005)
Somes River, Romania	172–314	80.9–106	Moldovan (2006)
River Tamar, UK	6–28	3–10	Sumner et al. (2010)
River Leça, Portugal	828 ± 40	462 ± 66	Homem et al. (2016)
Molgora River, Italy	< LOD-1141	< LOD-364	Villa et al. (2012)
Margorabbia River, Italy	57.48–256.05	< LOD-10.24	This study
Bardello River, Italy	159.02–486.79	7.85–28.07	This study
Boesio River, Italy	218.51–534.94	13.72–44.58	This study
Tresa River, Italy	26.1–93.77	< LOD	This study
Ticino Emissary, Italy	< LOD-12.8	< LOD	This study

Regarding water samples, Boesio and Bardello rivers were generally one order of magnitude more contaminated by HHCB respect to Chinese rivers as Suzhou Creek, Haie River, and Songhua River (Zhang et al. 2008; Hu et al. 2011; Lu et al. 2015), while Nakdong River in Korea resulted more contaminated by HHCB, reaching peaks of 13,920 ng L^−1^ (Lee et al. 2010). This might be due to a combination of both the river discharge and the usage of PCMs in that area. In fact, Chinese rivers considered in Zhang et al. (2008) are large rivers with a high water flow that is able to dilute PCM concentrations discharged by WWTP effluents, while Lee et al. (2010) evidenced how the predominance of HHCB in the analyzed samples was consistent with the high usage of HHCB in Korea, around the 80% of the total usage of PCMs in the Korean market. Considering the other tributaries of Lake Maggiore as Toce and Ticino Tributary, together with Ticino Emissary, our results were comparable, for example, to the low concentrations detected in the Hudson River located in the USA (Reiner and Kannan 2011). Again, this was probably due to the high mean water flow of Ticino and Toce Rivers, 350 m^3^ s^−1^ and 70 m^3^ s^−1^, respectively, comparable to the water flow of Hudson River, 606 m^3^ s^−1^. In this case, a strong dilution effect may be assumed. Regarding Europe, our results were almost in line with concentrations detected in other European Rivers (Moldovan 2006; Sumner et al. 2010; Lange et al. 2015). However, rivers located in densely populated area as Berlin (Fromme et al. 2001) and Molgora River in Italy (Villa et al. 2012) registered higher concentrations than our study probably because pollution caused by domestic discharges is here more intensive and the rivers are not able to dilute PCMs to reach lower concentration levels.

Considering sediments, concentrations from previously published studies are summarized in Table 4.

**Table 4:** Worldwide concentrations of HHCB and AHTN in sediments (ng g^−1^ d.w.)

Location	HHCB (ng g^−1^ d.w.)	AHTN (ng g^−1^ d.w.)	Reference
Asia			
Suzhou Creek, China	3–78	2–31	Zhang et al. (2008)
Haihe River, China	1.50–32.3	2–21.9	Hu et al. (2011)
Songhua River, China	< 0.5–17.5	< 0.5–5.7	Lu et al. (2015)
The North Canal River, China	4.10–818	1.21–731	Zhang et al. (2020)
Shanghai River, China	< LOD-61.7	< LOD–2.78	Wang et al. (2018)
Liangtan River, China	< LOD-268	< LOD–99.8	Sang et al. (2012)
Pearl River, China	5.65–31.5	4.54–18.9	Huang et al. (2016)
Yellow River, China	1.42–8.6	< LOD-3.63	Lou et al. (2016)
Urban rivers, Guangzhou, China (Dry season)	< LOD-1480	< LOD-235	Peng et al. (2017)
Urban rivers, Guangzhou, China (Wet season)	< LOD-1430	< LOD-205	Peng et al. (2017)
Urban catchment, Singapore	11–108	3.5–27.3	Wang and Kelly (2017)
Nakdong River, Korea	< LOD-6.30	< LOD-2.30	Lee et al. (2014)
America			
Hudson River, USA	72.8–388	113–544	Reiner and Kannan (2011)
Europe			
Lippe River, Germany	< LOD-56	< LOD–90	Kronimus et al. (2004)
Berlin, Germany	220–920	20–1100	Fromme et al. (2001)
Molgora River, Italy	< LOD-18,000	< LOD-4320	Villa et al. (2012)
Toce River, Italy	< LOD-85.42	< LOD-78.87	This study
Margorabbia River, Italy	< LOD-12.38	< LOD-4.89	This study
Tresa River, Italy	< LOD-20.20	< LOD	This study
Ticino Emissary, Italy	20.62–78.64	< LOD-21.02	This study
Bardello River, Italy	< LOD-238.53	11.89–128.39	This study
Boesio River, Italy	26.57–394.80	7.55–79.74	This study
Ticino Tributary, Switzerland	< LOD	< LOD	This study

When compared to studies conducted worldwide, our results were similar to that reported for most regions and countries in terms of relative contribution of HHCB and AHTN. In fact, worldwide, HHCB is the main polycyclic musk detected in sediment samples. Regarding absolute concentrations, depending on the considered river, our result can be comparable to rivers with high water flows which can dilute fragrance concentration or to rivers which cross heavy anthropized areas that can impact on freshwater ecosystems by discharging these chemicals through WWTP effluents. For example, data of Ticino Tributary, Toce, Tresa, and Margorabbia rivers of this study are comparable to other emissaries and large rivers as Suzhou Creek River in China (Zhang et al. 2008), which is one of the emissaries of Lake Tai, and Songhua and Lippe Rivers, characterized by high water flow, 2470 m^3^ s^−1^ and 241 m^3^ s^−1^, respectively (Kronimus et al. 2004; Lu et al. 2015). On the contrary, concentrations of synthetic musks detected in Boesio and Bardello sediments are comparable to that measured in other anthropized rivers as Hudson River in the USA (Reiner and Kannan 2011), the North Canal River watershed in China, an urban catchment located in the megacity of Beijing (Zhang et al. 2020), and rivers crossing other urban catchments as Singapore and Berlin (Fromme et al. 2001; Wang and Kelly 2017). Besides synthetic musk fragrances, the presence of other persistent organic contaminants, including those of industrial origin, such as brominated and chlorinated compounds, is widely documented in these two rivers (Marziali et al. 2021), which therefore appear to be the main contamination sources of Lake Maggiore.

### Potential environmental risk assessment of HHCB and AHTN

The environmental risk assessment of HHCB and AHTN in surface water and sediment was performed according to the Hazard Quotient (*HQ*) approach already employed in other studies (Raybould et al. 2011). Considering for water HHCB and AHTN *PNEC* values of 4400 ng L^−1^ and 2800 ng L^−1^, respectively (European Commission 2008b, a) our data showed *HQ* for AHTN always < 0.1 in all samples, thus indicating a low risk level for this compound. Similarly, HHCB expressed low risk (*HQ* < 0.1) in most of the analyzed samples even if sometimes exceeding the value of 0.1 in winter samples of Bardello and Boesio rivers and even in spring only in Boesio River, thus posing a medium risk for the environment. Therefore, exposure of aquatic organisms to polycyclic musk fragrances seems to be higher in winter, probably because of the lower river dilution factor that can mitigate discharge impact. Similarly, even in Indian (Vimalkumar et al. 2021) and Chinese (Zeng et al. 2018; Wang et al. 2018) rivers characterized by the presence of important discharges of domestic and industrial wastewaters, synthetic musks do not pose any health hazard to aquatic organisms. The combined ecological risk was calculated on the sum of *HQs* of HHCB and AHTN (Guo et al. 2013). A combined *HQ* ≤ 0.01, between 0.01 and 0.1, between 0.1 and 1, and ≥ 1.0 can be traduced into an ecological risk level of no, probable low, medium, and high risks (Lee et al. 2014). In water samples, combined *HQ* resulted in a probable low risk in Margorabbia, Bardello, and Boesio rivers, sometimes with peaks reaching the expected medium risk range in winter samples in Boesio and Bardello. No ecological risk resulted from combined *HQ* of Tresa and Ticino Emissary water samples.

Regarding sediments, in the present study measured concentrations of synthetic musks were lower than their respective *PNEC* values for sediment-dwelling organisms, and the resulting *HQ* of AHTN was below 0.1 in all rivers indicating that the risk caused by this chemical was low in all sampling sites. Regarding HHCB, *HQ* was again below 0.1 in all tributaries except in Boesio and Bardello rivers in which a medium risk regarding HHCB was evidenced. Considering again the combined ecological risk for sediments, Ticino Emissary showed a probable low risk level, while Boesio and Bardello rivers expressed an expected medium risk level, with a combined *HQ* between 0.1 and 1. Therefore, even for sediment-dwelling organisms, Boesio and Bardello rivers expressed a medium risk level. In general, since field-derived log *K*_OC_ values for HHCB and AHTN are in the range of 3.86–4.86 (Fooken 2004; Wang et al. 2018), these compounds are expected to accumulate primarily in suspended particulates, sediments, and within organisms resulting in a biomagnification hazard as already evidenced in other aquatic ecosystems (Zhang et al. 2013; Hu et al. 2011).

However, this preliminary approach does not take into consideration other pollutants which may be present in surface waters or sediments, and which might contribute to increase in the combined ecological risk with synergistic and additive effects. In addition, *HQ* approach does not take into consideration processes such as bioaccumulation and contaminant metabolisms in aquatic organisms together with long-term exposure effects (Guo et al. 2013). Further data are necessary to evaluate potential adverse effects of polycyclic musk under the contest of long-term exposure and pollutant mixtures.

## Conclusions

In this study, the presence of synthetic musk fragrances was investigated in surface waters and sediments of the main tributaries of a large deep subalpine lake in Northern Italy. A wide range of PCM concentration was measured in the considered rivers, from few ng L^−1^ to values above 500 ng L^−1^. HHCB was the main detected compound in all samples, as expected from its extensive usage in commercial products. Once released in surface waters, synthetic musks may be accumulated in sediments which therefore may represent their final sink. Levels of synthetic musks were strictly dependent on anthropogenic pressures along the river courses, particularly the presence of WWTP which can discharge these chemicals through their effluents.

Chemical data gathered during the study, in particular, N and P concentrations, confirmed this hypothesis since high PCM values were detected in the more polluted rivers located in areas where intensive anthropogenic pressures are present. The ecological risk posed by individual and combined PCMs in freshwaters and sediments are still acceptable; however, Boesio and Bardello rivers showed an expected medium risk levels during dry seasons, where dilution does not compensate the input of these chemicals from WWTPs. A site-specific ecological risk assessment together with bioaccumulation studies is needed to fully understand the potential impact of these chemicals on aquatic organisms.

This study provides the first valuable information regarding the presence of synthetic musks in the studied ecosystem. However, more data are needed to identify punctual emission sources of these compounds, alongside with bioaccumulation studies to evaluate the effective bioavailability and further investigate the ecological risk posed by synthetic musks in the environment.

### Supplementary Information

Below is the link to the electronic supplementary material.

Supplementary file1 (DOCX 92 KB)

## Data Availability

Not applicable.
