# Supplementary material for: Behaviour of synthetic musk fragrances in freshwaters: occurrence, relations with environmental parameters, and preliminary risk assessment
Source: Environ Sci Pollut Res Int. 2023 Sep 30;30(50):109643–58. doi: 10.1007/s11356-023-30030-9 (PMC10622367; doi:10.1007/s11356-023-30030-9)
Supplement: Supplementary file 1 — Supplementary file1 (DOCX 92 KB) [file 11356_2023_30030_MOESM1_ESM.docx]

**Supplementary Material**

**Behaviour of synthetic musk fragrances in freshwaters: occurrence, relations with environmental parameters and preliminary risk assessment**

Stefano Tasselli^1*^, Michela Rogora^2^, Arianna Orrù^2^ and Licia Guzzella^1^

^1^National Research Council - Water Research Institute (CNR-IRSA), Via del Mulino 19, Brugherio (MB), 20861, Italy

^2^National Research Council - Water Research Institute (CNR-IRSA), L.go Tonolli 50, Verbania (VB), 28922, Italy

*Corresponding author: stefano.[tasselli@irsa.cnr.it](mailto:tasselli@irsa.cnr.it); +39 03921694207

**Tables**

**Table S1.** Main validation parameters for PMF analyses in water samples**.**

| Compound | Linearity range (ng L^-1^) | R^2^ | LOD^a^  (ng L^-1^) | Intra-day precision  (%RSD^b^)  *n*=3 | | Inter-day precision  (%RSD)  *n*=3 | Accuracy^f^  (%Recovery tests)  *n*=3 | |
| --- | --- | --- | --- | --- | --- | --- | --- | --- |
|  |  |  |  | [C_1_]^c^ | [C_2_]^d^ | [C_3_]^e^ | [C_1_]^c^ | [C_2_]^d^ |
| ADBI | 0.5-400 | 0.992 | 0.5 | 5 | 0.4 | 7 | 81 | 81 |
| AHDI | 0.5-400 | 0.994 | 0.5 | 10 | 2 | 4 | 104 | 95 |
| AHTN | 0.5-400 | 0.999 | 5 | 7 | 3 | 4 | 93 | 90 |
| HHCB | 2.5-2000 | 0.998 | 2.5 | 5 | 3 | 5 | 86 | 94 |
| HHCB-L | 2.5-2000 | 0.996 | 2.5 | 13 | 4 | 2 | 103 | 89 |

^a^ LOD – limit of detection (*S/N*=3).

^b^ RSD – Relative standard deviation, three replicate analysis of a spiked real water sample.

^c^ [C_1_] – low concentration level: spike of 150 ng L^-1^ of HHCB and HHCB-L and 30 ng L^-1^ of ADBI, AHDI and AHTN.

^d^ [C_2_] – high concentration level: spike of 1500 ng L^-1^ of HHCB and HHCB-L and 300 ng L^-1^ of ADBI, AHDI and AHTN.

^e^ [C_3_] – concentration level for inter-day precision evaluation: spike of 150 ng L^-1^ of HHCB and HHCB-L and 30 ng L^-1^ of ADBI, AHDI and AHTN.

^f^ Accuracy – average recovery percentage, three replicate analyses of a spiked real water sample.

**Table S2.** Main validation parameters for PMF analyses in sediment samples**.**

| Compound | Linearity range  (ng g^-1^) | R^2^ | LOD^a^  (ng g^-1^) | Intra-day precision  (%RSD^b^)  *n*=3 | | | Inter-day precision  (%RSD) *n*=3 | Accuracy^g^  (%Recovery tests)  *n*=3 | | |
| --- | --- | --- | --- | --- | --- | --- | --- | --- | --- | --- |
|  |  |  |  | [C_1_]^c^ | [C_2_]^d^ | [C_3_]^e^ | [C_4_]^f^ | [C_1_]^c^ | [C_2_]^d^ | [C_3_]^e^ |
| ADBI | 0.625-500 | 0.992 | 0.625 | 14 | 10 | 7 | 4 | 95 | 98 | 80 |
| AHDI | 0.625-500 | 0.994 | 0.625 | 9 | 5 | 9 | 3 | 82 | 90 | 86 |
| AHTN | 0.625-500 | 0.999 | 6.250 | 19 | 6 | 7 | 5 | 86 | 91 | 90 |
| HHCB | 3.125-2500 | 0.998 | 3.125 | 39 | 3 | 8 | 4 | 86 | 91 | 95 |
| HHCB-L | 3.125-2500 | 0.996 | 3.125 | 23 | 12 | 7 | 3 | 82 | 120 | 97 |

^a^ LOD – limit of detection (*S/N*=3).

^b^ RSD – Relative standard deviation, three replicate analysis of a spiked real sediment sample.

^c^ [C_1_] – low concentration level: spike of 50 ng g^-1^ of HHCB and HHCB-L and 10 ng g^-1^ of ADBI, AHDI and AHTN.

^d^ [C_2_] – medium concentration level: spike of 500 ng g^-1^ of HHCB and HHCB-L and 100 ng g^-1^ of ADBI, AHDI and AHTN.

^e^ [C_3_] – high concentration level: spike of 5000 ng g^-1^ of HHCB and HHCB-L and 1000 ng g^-1^ of ADBI, AHDI and AHTN.

^f^ [C_4_] – concentration level for inter-day precision evaluation: spike of 250 ng g^-1^ of HHCB and HHCB-L and 50 ng g^-1^ of ADBI, AHDI and AHTN.

^g^ Accuracy – average recovery percentage, three replicate analyses of a spiked real sediment sample.

**Figures**

**Figure S1.** Monthly discharge (m^3^ s^-1^) of the main tributaries of Lake Maggiore during the present study. Data from CNR IRSA (2021, 2022). (2022). Ticino Emissary is represented on the right scale.

**b**

**c**

**d**

**f**

**g**

**h**

**i**

**Figure S2.** Monthly chemical parameters of Lake Maggiore main tributaries and Ticìno Emissary. a= conductivity at 20°C; b=alkalinity; c=nitrate; d=ammonium; e= organic nitrogen obtained by difference between total nitrogen and inorganic nitrogen; f=total nitrogen; g=total phosphorus; h=reactive silica; i= total organic carbon.
